# Supplementary material for: Strategies to Increase Flu Vaccination Coverage among Healthcare Workers: A 4 Years Study in a Large Italian Teaching Hospital
Source: Vaccines (Basel). 2020 Feb 13;8(1):85. doi: 10.3390/vaccines8010085 (PMC7157643; doi:10.3390/vaccines8010085)
Supplement: Supplementary file 1 [file vaccines-08-00085-s001.pdf]

Table S1. Vaccination coverage rates in the 36 clinical macro-areas randomized for the first On Site Vaccination, during the influenza vaccination campaign 2016-17. Macro-areas randomly selected for OSV = 12/36 (33.33%); HCWs potentially involved in OSV = 1120/3654 (30.65%).

**Macro-areas***	**Randomly selected for OSV**	**Vaccinated**	**n. HCWs operating in macro-areas****	**%**
Health Management	No	23	54	42.59
Pediatrics	Yes	31	107	28.97
Infectious Disease	Yes	17	63	26.98
Hematology	Yes	23	97	23.71
Pneumology and Thoracic Surgery	Yes	11	57	19.30
Orthopedics	Yes	9	64	14.06
Cardiac Surgery	Yes	5	36	13.89
Oncology	Yes	13	95	13.68
Medicine (CIC)	No	8	70	11.43
Transplant Surgery	Yes	4	35	11.43
Orthopedics (CIC)	No	4	36	11.11
Intensive Care	Yes	25	235	10.64
Emergency Room	No	11	107	10.28
General Surgery (CIC)	No	8	79	10.13
Radiology	No	19	198	9.68
Neurology	No	14	147	9.52
Medicine	No	18	201	8.96
General Surgery	No	10	119	8.40
Urology	No	5	60	8.33
Geriatrics (CIC)	No	1	12	8.33
Laboratory	No	13	159	8.18
Cardiology	Yes	8	123	6.50
Allied and Technical Service	No	16	261	6.13
Geriatrics	Yes	8	133	6.02
Private Activity	No	3	51	5.88
Cardiology (CIC)	No	2	34	5.88
Intensive Care (CIC)	No	2	38	5.26
Operating Room (CIC)	No	2	47	4.26
Head and Neck Surgery	No	6	144	4.17
Rheumatology (CIC)	Yes	3	75	4.00
Radiology (CIC)	No	1	27	3.70
Operating Room	No	9	314	2.87
Neurosurgery	No	2	71	2.82
Gynecology and Obstetrics	No	5	223	2.24
Allied and Technical Service (CIC)	No	1	61	1.64
Private Activity (CIC)	No	0	21	0.00
**Total**		**340**	**3654**	**9.30**

*(CIC) = macro-areas located in Presidio Columbus **Personnel not included: administrative, staff (e.g. technicians, clerks).

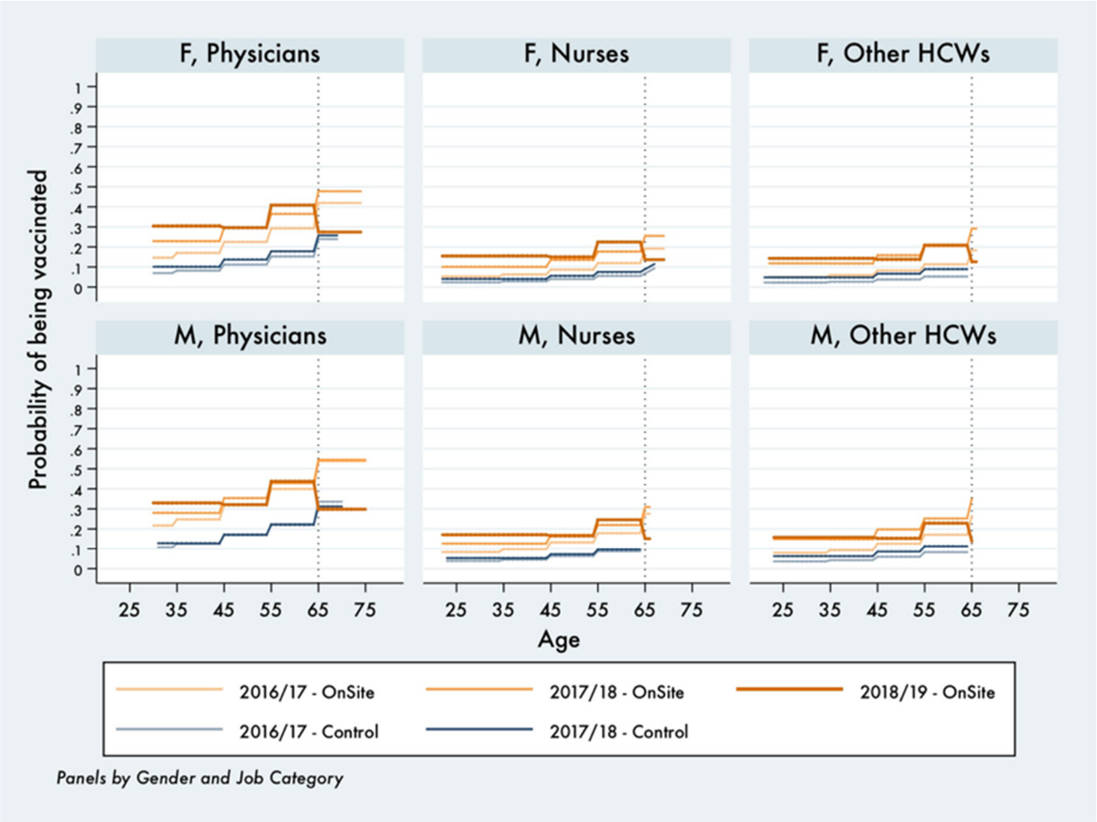

Figure S1: Probability of being vaccinated by age class, gender, OSV and job category during the three campaigns (model without interaction, available in table 3 in main text).

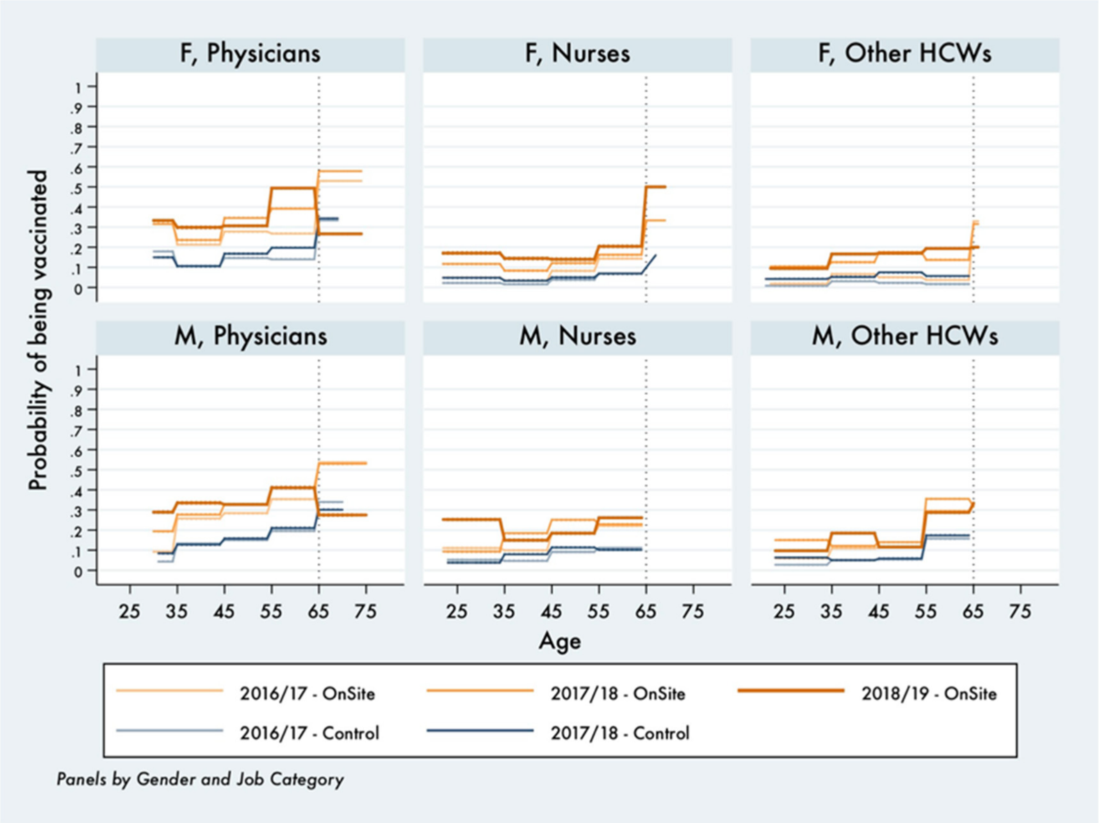

Figure S2: Probability of being vaccinated by age class, gender, OSV and job category during the three campaigns (model with multiple interaction, available in supplementary material).

Table S2. Multivariate logistic regression model (non linear age + OSV + gender + job category with multiple interaction) by campaign

The 2016-17 influenza season

Logistic regression	Number of obs	=	3,554
	LR chi2(26)	=	271.65
	Prob > chi2	=	0.0000
Log likelihood = -962.67934	Pseudo R2	=	0.1236

vacc	Odds Ratio	Std. Err.	z	P>|z|	[95% Conf.	Interval]
1.rep_onsite	2.25191	.2797474	6.53	0.000	1.765262	2.872718
ageclas						
35/44	.5502647	.2743219	-1.20	0.231	.2071212	1.461904
45/54	.7844346	.3979097	-0.48	0.632	.2902546	2.119993
55/64	.7437376	.4008029	-0.55	0.583	.258645	2.138629
65/75	2.290886	2.985381	0.64	0.525	.1781375	29.46128
professione3						
infermieri	.1043988	.0560979	-4.21	0.000	.0364174	.299283
OHCWs	.0388687	.0426943	-2.96	0.003	.0045146	.3346408
ageclas#professione3						
35/44#infermieri	1.28032	.854841	0.37	0.711	.3459317	4.738564
35/44#OHCWs	6.784419	8.222955	1.58	0.114	.6307052	72.97917
45/54#infermieri	2.215389	1.382872	1.27	0.203	.6518083	7.529739
45/54#OHCWs	3.554508	4.513856	1.00	0.318	.2950137	42.82693
55/64#infermieri	4.381665	2.928592	2.21	0.027	1.182274	16.23904
55/64#OHCWs	2.721783	4.149283	0.66	0.511	.1371578	54.01154
65/75#infermieri	1	(empty)				
65/75#OHCWs	1	(empty)				
sex						
M	.2071989	.1768297	-1.84	0.065	.0388998	1.103642
ageclas#sex						
35/44#M	6.193781	5.613682	2.01	0.044	1.048278	36.59614
45/54#M	4.968021	4.535375	1.76	0.079	.8300607	29.73426
55/64#M	7.225502	6.663495	2.14	0.032	1.185425	44.04151
65/75#M	4.964457	7.61194	1.05	0.296	.2458868	100.2325
professione3#sex						
infermieri#M	11.71335	11.48298	2.51	0.012	1.714825	80.0096
OHCWs#M	16.03724	24.10879	1.85	0.065	.8423986	305.3103
ageclas#professione3#sex						
35/44#infermieri#M	.2019916	.2313041	-1.40	0.162	.0214095	1.90572
35/44#OHCWs#M	.0837038	.1395486	-1.49	0.137	.0031889	2.197069
45/54#infermieri#M	.2101029	.2310443	-1.42	0.156	.0243438	1.813322
45/54#OHCWs#M	.146599	.2493905	-1.13	0.259	.005225	4.11314
55/64#infermieri#M	.096655	.1092412	-2.07	0.039	.0105483	.8856613
55/64#OHCWs#M	.4532179	.8490142	-0.42	0.673	.0115279	17.81825
65/75#infermieri#F	1	(empty)				
65/75#infermieri#M	1	(empty)				
65/75#OHCWs#F	1	(empty)				
65/75#OHCWs#M	1	(empty)				

_cons	.2182562	.0971705	-3.42	0.001	.0912012	.5223152

Note: _cons estimates baseline odds.
note: 5.ageclas#1.professione3#1.sex != 0 predicts failure perfectly
 5.ageclas#1.professione3#1.sex dropped and 1 obs not used

note: 5.ageclas#1.professione3#2.sex != 0 predicts failure perfectly
 5.ageclas#1.professione3#2.sex dropped and 1 obs not used

note: 5.ageclas#2.professione3#1.sex identifies no observations in the sample
note: 5.ageclas#2.professione3#2.sex identifies no observations in the sample

The 2017-18 influenza season

Logistic regression   Number of obs   =   3,663
                                                  LR chi2(27)   =   278.13
                                                  Prob > chi2   =   0.0000
Log likelihood = -1346.4562   Pseudo R2   =   0.0936

vacc	Odds Ratio	Std. Err.	z	P>|z|	[95% Conf.	Interval]
1.rep_onsite	2.618295	.2792614	9.02	0.000	2.124376	3.227051
ageclas						
35/44	.670469	.3804721	-0.70	0.481	.2204683	2.038973
45/54	1.145955	.6540401	0.24	0.811	.374416	3.507363
55/64	1.395336	.826212	0.56	0.574	.4371816	4.453441
65/75	2.973572	2.9453	1.10	0.271	.4267466	20.71987
professione3						
infermieri	.2876309	.1614169	-2.22	0.026	.0957516	.864023
OHCWs	.2525542	.166714	-2.08	0.037	.0692574	.9209645
ageclas#professione3						
35/44#infermieri	1.027372	.6497559	0.04	0.966	.2974334	3.54867
35/44#OHCWs	1.842813	1.401396	0.80	0.421	.4151185	8.180702
45/54#infermieri	.9059028	.5613404	-0.16	0.873	.2689285	3.051592
45/54#OHCWs	1.597637	1.206091	0.62	0.535	.3638207	7.015664
55/64#infermieri	1.052272	.6891813	0.08	0.938	.291499	3.79856
55/64#OHCWs	.9787439	.8338045	-0.03	0.980	.1842975	5.197789
65/75#infermieri	1.268191	2.011916	0.15	0.881	.0565977	28.41646
65/75#OHCWs	1	(empty)				
sex						
M	.5221403	.3772584	-0.90	0.368	.1266994	2.15179
ageclas#sex						
35/44#M	2.369235	1.835638	1.11	0.266	.5189354	10.8169
45/54#M	1.782121	1.381924	0.75	0.456	.3898374	8.146871
55/64#M	2.070052	1.622568	0.93	0.353	.4454349	9.620068
65/75#M	1.574585	1.817219	0.39	0.694	.1639833	15.11933
professione3#sex						
infermieri#M	1.486696	1.238437	0.48	0.634	.2905094	7.608236
OHCWs#M	2.90767	2.709377	1.15	0.252	.4681583	18.05916
ageclas#professione3#sex						
35/44#infermieri#M	1.34778	1.271362	0.32	0.752	.2121679	8.561663
35/44#OHCWs#M	.2663342	.2926129	-1.20	0.229	.03092	2.294113

45/54#infermieri#M	1.757753	1.640484	0.60	0.546	.282192	10.94891
45/54#OHCWs#M	.2814848	.3081852	-1.16	0.247	.032924	2.406566
55/64#infermieri#M	.9472328	.9194685	-0.06	0.955	.1413204	6.34905
55/64#OHCWs#M	1.100662	1.23278	0.09	0.932	.1225402	9.886197
65/75#infermieri#M	1	(empty)				
65/75#OHCWs#F	1	(empty)				
65/75#OHCWs#M	1	(empty)				
_cons	.1760568	.0943241	-3.24	0.001	.0616046	.503144

Note: _cons estimates baseline odds.

note: 5.ageclas#1.professione3#2.sex != 0 predicts failure perfectly
5.ageclas#1.professione3#2.sex dropped and 1 obs not used

note: 5.ageclas#2.professione3#1.sex identifies no observations in the sample
note: 5.ageclas#2.professione3#2.sex identifies no observations in the sample

The 2018-19 influenza season

Logistic regression

Number of obs	=	4,247
LR chi2(28)	=	209.13
Prob > chi2	=	0.0000
Pseudo R2	=	0.0468

Log likelihood = -2131.5149

vacc	Odds Ratio	Std. Err.	z	P>|z|	[95% Conf.	Interval]
ageclas						
35/44	.852071	.2278875	-0.60	0.549	.5044532	1.439232
45/54	.8837209	.2646266	-0.41	0.680	.49139	1.589293
55/64	1.952381	.6171621	2.12	0.034	1.050736	3.627736
65/75	.7272727	.4557124	-0.51	0.611	.212976	2.483499
professione3						
infermieri	.4111498	.1104438	-3.31	0.001	.242856	.6960674
OHCWs	.2077922	.0721963	-4.52	0.000	.1051681	.410558
ageclas#professione3						
35/44#infermieri	.9631617	.3240763	-0.11	0.911	.4980744	1.862534
35/44#OHCWs	2.259201	.9598913	1.92	0.055	.9824167	5.195342
45/54#infermieri	.8963202	.315349	-0.31	0.756	.4497675	1.786233
45/54#OHCWs	2.231854	1.015084	1.77	0.078	.9152195	5.442598
55/64#infermieri	.639418	.2409711	-1.19	0.235	.3054928	1.338347
55/64#OHCWs	1.183171	.5769778	0.34	0.730	.4549416	3.077082
65/75#infermieri	6.688559	7.950856	1.60	0.110	.6508609	68.73485
65/75#OHCWs	3.308594	4.328605	0.91	0.360	.2546969	42.97968
sex						
M	.8125	.2642945	-0.64	0.523	.4294769	1.537117
ageclas#sex						
35/44#M	1.454545	.5567293	0.98	0.328	.6869557	3.079824
45/54#M	1.355072	.5608129	0.73	0.463	.6021187	3.049597
55/64#M	.8775084	.3655164	-0.31	0.754	.3878758	1.985226
65/75#M	1.282051	.908242	0.35	0.726	.3198083	5.139502
professione3#sex						
infermieri#M	2.021572	.8521655	1.67	0.095	.8848648	4.618504
OHCWs#M	1.27574	.736231	0.42	0.673	.4116542	3.95359

```
ageclas#professione3#sex |
     35/44#infermieri#M  |   .4362081   .2440891    -1.48   0.138     .1456755    1.306173
          35/44#OHCWs#M  |   .7517007   .5239795    -0.41   0.682     .1917377    2.947015
     45/54#infermieri#M  |   .6240956   .3538026    -0.83   0.406     .2054476    1.895837
          45/54#OHCWs#M  |   .4531746   .3428342    -1.05   0.295     .1028768    1.996245
     55/64#infermieri#M  |   .9541551   .5581736    -0.08   0.936     .3031604     3.00307
          55/64#OHCWs#M  |   1.848439    1.34651     0.84   0.399     .4433437    7.706719
     65/75#infermieri#M  |          1  (empty)
          65/75#OHCWs#M  |   1.505009   2.807198     0.22   0.827     .0388908    58.24132
                         |
                   _cons |         .5   .1137147    -3.05   0.002     .3201708    .7808332
------------------------------------------------------------------------------------------
Note: _cons estimates baseline odds.
note: 5.ageclas#1.professione3#2.sex != 0 predicts failure perfectly
      5.ageclas#1.professione3#2.sex dropped and 1 obs not used
```
